# Hysteretic photochromic switching of Eu-Mg defects in GaN links the shallow transient and deep ground states of the Mg acceptor

**DOI:** 10.1038/srep41982

**Published:** 2017-02-03

**Authors:** A. K. Singh, K. P. O’Donnell, P. R. Edwards, K. Lorenz, M. J. Kappers, M. Boćkowski

**Affiliations:** 1 SUPA Department of Physics, University of Strathclyde, 107 Rottenrow, Glasgow G4 0NG, Scotland, United Kingdom; 2 IPFN, Instituto Superior Técnico, Universidade de Lisboa, Campus Tecnológico e Nuclear, Estrada Nacional 10, 2695-066 Bobadela LRS, Portugal; 3 Department of Materials Science and Metallurgy, University of Cambridge, 27 Charles Babbage Road, Cambridge CB3 0FS, England, United Kingdom; 4 Institute of High Pressure Physics PAS, Sokolowska 29/37, 01-142 Warsaw, Poland

## Abstract

Although p-type activation of GaN by Mg underpins a mature commercial technology, the nature of the Mg acceptor in GaN is still controversial. Here, we use implanted Eu as a ‘spectator ion’ to probe the lattice location of Mg in doubly doped GaN(Mg):Eu. Photoluminescence spectroscopy of this material exemplifies hysteretic photochromic switching (HPS) between two configurations, Eu0 and Eu1(Mg), of the same Eu-Mg defect, with a hyperbolic time dependence on ‘switchdown’ from Eu0 to Eu1(Mg). The sample temperature and the incident light intensity at 355 nm tune the characteristic switching time over several orders of magnitude, from less than a second at 12.5 K, ~100 mW/cm^2^ to (an estimated) several hours at 50 K, 1 mW/cm^2^. Linking the distinct Eu-Mg defect configurations with the shallow transient and deep ground states of the Mg acceptor in the Lany-Zunger model, we determine the energy barrier between the states to be 27.7(4) meV, in good agreement with the predictions of theory. The experimental results further suggest that at low temperatures holes in deep ground states are localized on N atoms axially bonded to Mg acceptors.

The achievement of p-type doping of GaN by Mg triggered the development of UV and blue light emitting and laser diodes^1^,^2^,^3^. Remarkably, GaN:Mg is one of the very few host-dopant combinations of a p-type wide-gap semiconductor that has matured into a commercial technology. Despite this technological success, the physics of the Mg acceptor states in GaN is still not settled^4^,^5^,^6^,^7^,^8^,^9^,^10^,^11^,^12^,^13^. Employing a Koopmans corrected density functional method, Lany and Zunger (L-Z, hereafter) proposed that GaN:Mg has two acceptor states with distinct properties: firstly, a highly non-effective-mass-like acceptor state (the deep ground state, DGS) with a hole localized in one p-orbital of a N neighbour in the basal plane; secondly, an effective-mass-like shallow transient state (STS)^7^. Lyons *et al*. later proposed that Mg_Ga_ does not exhibit conventional shallow acceptor behaviour, but is characterized by a highly localized hole with most of the charge located on an axial N nearest neighbour^8^. On the experimental side, Callsen *et al*. recorded excitation-wavelength- and temperature-dependent photoluminescence (PL) spectra of GaN:Mg and determined donor and acceptor binding energies, localization and activation energies^9^. Based on these findings, they endorsed the L-Z model with experimentally determined binding energies of 164 ± 5 and 195 ± 5* *meV for the STS and DGS states, respectively. More recently, Davies proposed, on the basis of an analysis of magnetic resonance studies, that STS can form only in strain-free regions of material, whereas DGS occurs in strained regions; for DGS, the hole is localized in a p-like orbit of a basal N atom^10^. Since there is only a small difference between parallel and perpendicular components of the optically detected magnetic resonance *g*-values, as expected for a deep acceptor, Alves *et al*. concluded that the hole spreads equally among all four N neighbours^11^.

Thus, there is strong disagreement about the existence, or not, of two Mg acceptor states in GaN, the influence of strain and the hole localization in the DGS state^7^,^8^,^9^,^10^,^11^,^12^,^13^. In this manuscript, we introduce a different experimental approach to address these important issues.

Although the *4 f* electrons of rare earth (RE) ions are strongly localized, the weak perturbation caused by the local crystal field of a given host crystal determines the spectral fine structure of the sharp intra-*4f* transitions by splitting the ground state ^7^F_J_ and excited state ^5^D_J_ multiplets^14^,^15^,^16^,^17^,^18^,^19^; in particular, the energy level shifts and splitting of Eu^3+^ in GaN are very sensitive probes of the local crystal field symmetry^19^. The PL spectrum of Eu^3+^ implanted into Mg-doped GaN reveals a set of emission peaks completely different from those observed most frequently in n-type GaN, viz. Eu2 (Eu_Ga_, the unassociated ‘prime’ defect^17^) and Eu1 (Eu_Ga_-X, where X is a lattice defect^15^). The spectrum dominant at room temperature in GaN(Mg):Eu has been labelled Eu0. Its associated defect centre, an RE ‘site’, also called Eu0, comprises a single Mg atom in close association with an Eu2 defect^19^.

Upon cooling a sample of GaN(Mg):Eu, the PL spectrum shows a striking transformation: as the temperature decreases below 40 K, the intensity of Eu0 *decreases* rapidly, contrary to normal behaviour, while an Eu1-like spectrum rises to replace it (see Fig. 1); this *photochromic* transformation was attributed to the structural instability of the Mg acceptor in GaN, an actual displacement of the associated Mg atom leading to a switch from a low-symmetry Eu0 spectrum to a more symmetric one, Eu1(Mg)^18^,^19^. In the present study, we use Eu^3+^ as a ‘spectator ion’ to probe the nature of Mg acceptor states in GaN. We investigate the dynamics of the transformation of Eu0 to Eu1(Mg), at a number of fixed temperatures below 50 K, as a function of excitation density. These studies allow a ‘dynamic’ interpretation of the temperature-dependent switching described in previous work^18^,^19^.

## Results

To study the photochromic dynamics systematically, we cool a sample *in the dark* to a set temperature between 12.5 and 45 K, allow it to stabilize for 30 min and then illuminate the sample. Despite the low temperature, the initial spectrum will be dominated by the unstable configuration Eu0. The return to equilibrium, with the spectrum dominated by Eu1(Mg), is monitored by recording a kinetic series of spectra, with a short acquisition time of 0.1 or 0.25 s, for time periods up to 1000 s after light onset. In this experiment, the exciting light simultaneously *induces* the observed switching and *monitors* it by PL spectroscopy.

The ^5^D_0_ and ^7^F_0_ levels of Eu^3+^ are singlets and the ^5^D_0_ → ^7^F_0_ (hereafter, 0-0) transition of any particular defect will show no line splitting. Figure 2 shows, in 3D representation, a plot of PL signal vs. wavelength vs. time, in the narrow 0-0 wavelength region from 586.5 nm to 589.5 nm, monitored at 18.5 K under 10 mW/cm^2^ laser excitation. The initial spectrum (*t* = 0.1 s) shows only a peak at ~587 nm corresponding to the 0-0 transition of the Eu0 defect, which provides the first indication of a substantial energy barrier to the transformation between the different configurations of the Eu-Mg defect *in the dark*. This peak fades rapidly with illumination time while a new peak, corresponding to the 0-0 transition of Eu1(Mg), appears at 588.9 nm. With further passage of time, the Eu1(Mg) defect signal completely replaces that of Eu0 within the noise level. When Eu0 and Eu1(Mg) are both present, a line with resonance behaviour (labelled ‘res’) appears near 588.6 nm, at first rising and then falling in intensity. The ‘res’ line is related to an intermediate metastable state and its intensity can be modelled as the product of Eu0 and Eu1(Mg)^18^. In time resolved photoluminescence switching, the intensity of ‘res’ line is found to be stronger at lower temperature, where the Eu0 to Eu1(Mg) transformation occurs on a shorter time scale. The inset to Fig. 2 compares the normalized PL signal of 0-0 transitions of Eu0 and Eu1(Mg) as a function of time, and shows that the decline of Eu0 is matched by the growth of Eu1(Mg). This confirms that Eu0 and Eu1(Mg) are two configurations of the same Eu-Mg defect.

Figure 3 compares the decay of the Eu0 PL signal as a function of (logarithmic) time under 10 mW/cm^2^ laser excitation at three different temperatures, 18.5 K, 30 K and 40 K. Attempts to fit these data using exponential functions gave extremely poor fits, especially towards longer times, where the exponential function shows a comparatively sharp decay. On the other hand, a hyperbolic function:


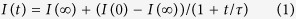


fits the data very well. *τ* is the time required to reduce the initial signal (*I*(0) − *I*(∞)) by a factor of 2 above the background level *I*(∞) ~ 0. At times much longer than the characteristic time *τ* of Eqn. (1), the signal decreases as 1/*t*. That *τ increases* with increasing temperature seems counterintuitive at first; we expect thermally activated processes to quicken at higher temperature. However, our experiments monitor a *return to equilibrium* from an unstable state. The increase of *τ* with temperature reflects the fact that the Eu0 defect is more stable at higher temperatures than is Eu1(Mg). By the same token, our determination of the activation energy by the standard fitting procedure for temperature dependences yields a *negative* value: the Arrhenius plot of Fig. 4 shows that *τ* is thermally activated above 25 K, with activation energies of 27.4(2), 27.2(5) and 28.5(9) meV measured using excitation densities of 1, 10 and 100 mW/cm^2^, respectively, and decreases with increasing excitation density. Below 25 K, *τ* is independent of temperature and averages 4.2, 1.1 and 0.33 s for excitation densities of 1, 10 and 100 mW/cm^2^, respectively. The measured activation energies agree within experimental error and average ~27.7(4) meV. This value is reasonably similar to the crossover energy barrier (~20 meV) *estimated* by L-Z for the STS to DGS transition^7^.

## Discussion

**Figure 1:**
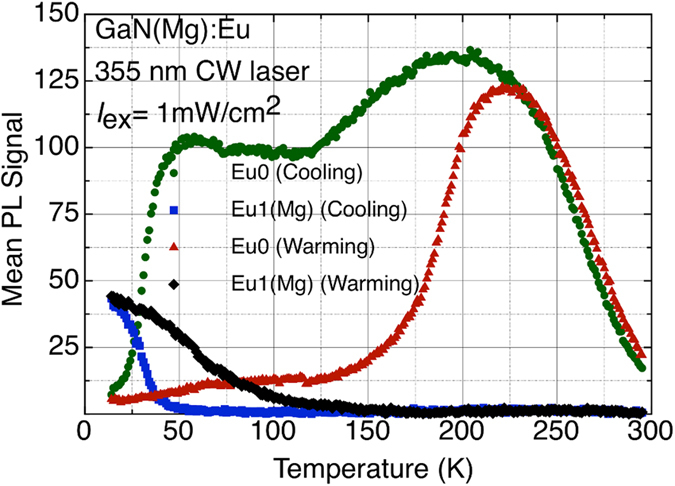
Mean PL Signal of ^5^D_0_ → ^7^F_0_ transition of Eu^3+^ for Eu0 and Eu1(Mg) configurations of Eu-Mg defect as a function of temperature under ~1 mW/cm^2^ excitation showing hysteretic photochromic switching between Eu0 and Eu1(Mg) configurations.

We proposed in earlier work that carrier freeze-out at low temperatures drives a lattice distortion, leading to the Eu0 to Eu1(Mg) transformation^18^. The transformation between Eu0 and Eu1(Mg) shows hysteretic behaviour: it occurs on sample cooling below 40 K, but it does not reverse on warming until the temperature rises above 130 K (see Fig. 1). During warming, the ionization of deep Mg acceptors above, say, 130 K results in a rapid increase in the mobile hole concentration: we suggested that it is the increase in the free hole population that triggers the reappearance of Eu0^18^,^19^. In light of the new results, it is clear that a state of dynamic equilibrium determines the balance of the Eu0 and Eu1(Mg) populations at all temperatures. At higher T, the photochromic switching from Eu0 to Eu1(Mg) is predicted to be *extremely* slow, taking many hours or even days at room temperature, dependent on excitation density. On lowering the temperature, switching is only observed at the point where the characteristic switching time *τ* is comparable in magnitude to the experimental data acquisition time. In this sense, cooling the sample *tunes* it into an experimental region where switching becomes observable. Further cooling decreases *τ* further towards a limiting value below 25 K and makes switching inevitable.

**Figure 2:**
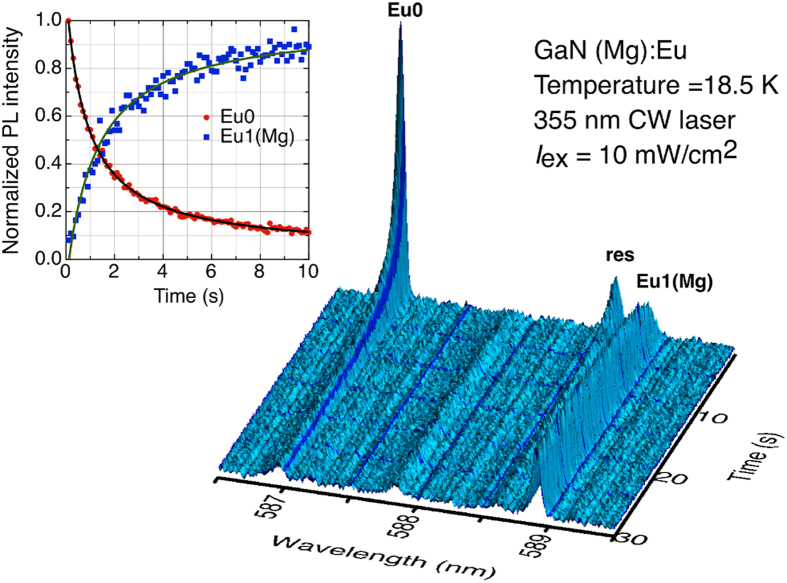
Showing a kinetic series of 300 PL spectra (^5^D_0_ → ^7^F_0_ transitions of Eu^3+^) under ~10 mW/cm^2^ excitation at 18.5 K (see text). The photochromic switching of Eu0 (587 nm) to Eu1(Mg) (588.9 nm) takes place in about 1 s. The inset shows that normalized PL signals of the Eu0 and Eu1(Mg) configurations are complementary in time.

**Figure 3:**
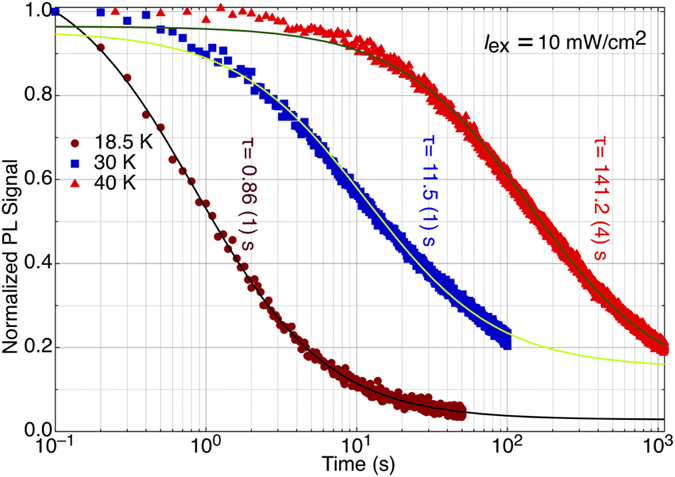
Showing the time evolution of mean Eu0 PL signal under 10 mW/cm^2^, 355 nm excitation at 18.5, 30 and 40 K. The solid lines show fits to Eqn. 1 with the characteristic times *τ* as indicated.

**Figure 4:**
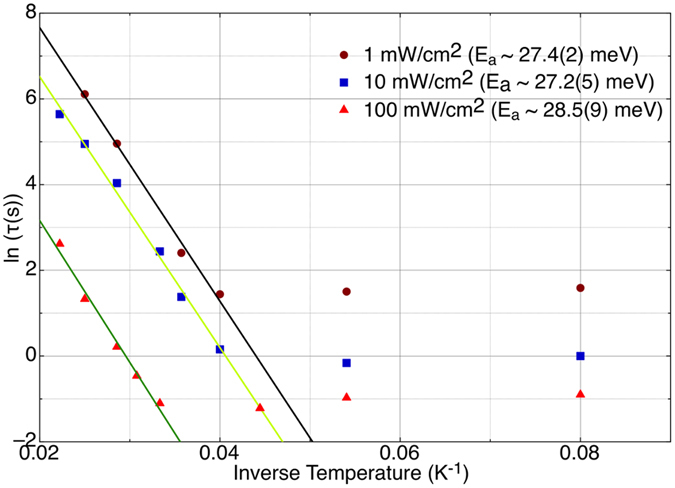
The Arrhenius plot (ln(*τ*) vs. inverse temperature) of Eu0/Eu1(Mg) switching at 3 different excitation densities.

It is important to mention at this point that two other models from the literature^20^,^21^ purport to explain temperature dependent switching of Eu-Mg defects; both of these models are incomplete in the sense that the authors have not recorded PL spectra during both a cooling and a warming run, which is essential to *observe* the hysteretic photochromic switching and *necessary* to *describe* its mechanism. In the first model, Lee *et al*.^20^ recorded PL spectra of GaN(Mg):Eu during a warming run and observed the anomalous growth of Eu0 emission. They describe the decrease in Eu1(Mg) (their peak B) PL intensity with increasing temperature as a typical temperature dependent PL behaviour of GaN:Eu and propose that the increase in PL intensity of Eu0 (peak A), up to 180 K, during warming, might be due to an increase in energy transfer efficiency from the host to the Eu0 defect. In the second model, Mitchell *et al*.^21^ propose that Eu0 (their Mg/Eu1) defect results from a coupling of a magnesium hydrogen (Mg-H) complex with Eu in GaN(Mg):Eu, and that under indirect or resonant excitation below 60 K, vibrational energy triggers H migration and modifies the complex which results in a transformation of the spectrum. They further report that such switching will not occur below a threshold excitation intensity nor at elevated temperatures, an observation consistent up to a point with our result that the characteristic switching time increases with increasing temperature and decreasing excitation intensities. Since, however, both models fail to reflect the 1:1 switching suggested by Fig. 2 and also neither acknowledge nor account for the reverse switching and the hysteresis observed in our experiments, we rule them out. Furthermore, we do not expect a significant presence of H in our samples after high temperature and high pressure annealing at 1673 K and 1 GPa of N_2_.

**Figure 5:**
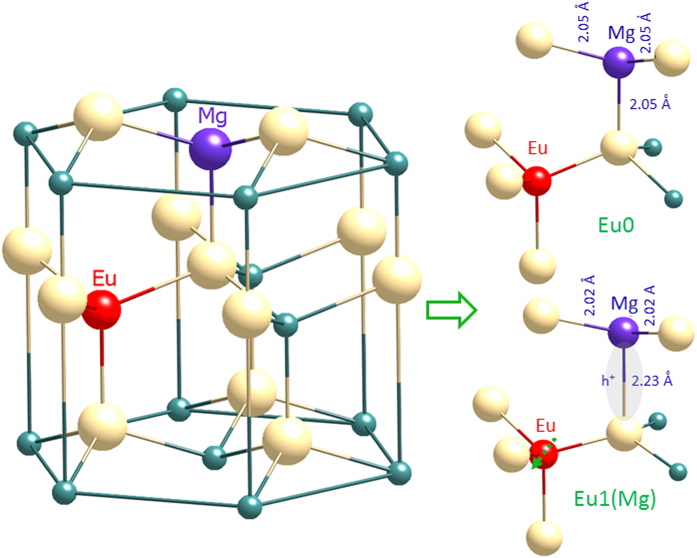
Showing the likely disposition of the Mg-Eu defect in Ga(Mg)N:Eu; on the right, the nanoscale phase changes that occur during the Eu0 to Eu1(Mg) transformation, with bond lengths taken from ref. 7.

We now extend our model^18^,^19^ in terms of the most likely disposition of Eu and Mg atoms in GaN in both configurations of the Eu0/Eu1(Mg) defect, according to the following considerations (see Fig. 5): (1) Eu and Mg impurities prefer Ga substitutional sites^22^,^23^,^24^; (2) the Eu0/Eu1(Mg) defect comprises a single Mg atom in close association with an Eu2 defect^18^; (3), Eu0 to Eu1(Mg) photochromic switching is related to the structural instability of the Mg acceptor in GaN^18^,^19^; (4) crystal field theory, applied to the ^7^F_1_ multiplet of Eu0, suggests a strong non-axial distortion^19^ which indicates that Mg might be linked through non-axial nitrogen to the spectator Eu^3+^ ion; (5) the emission lines, especially those of Eu1(Mg), are very sharp, indicating the presence of definite sites rather than a distribution. It is also important to mention that while there may be some interstitial Mg impurity in the crystal, as suggest by recent theory^13^, we do not believe it plays a part in the constitution of the Eu0/Eu1(Mg) defect. The larger atomic size of Eu results in a longer Eu–N bond (~2.25 Å) in comparison to Ga–N (~1.95 Å)^22^, which further diminishes the probability of occupation of interstitial sites by Mg in this case.

In the STS configuration of the Mg acceptor in GaN, all Mg–N distances are practically identical (~2.05 Å), whilst in DGS, a Jahn-Teller distortion displaces the N atom hosting the hole to a much larger separation (2.23 Å) from Mg than the other three N atoms (~2.02 Å)^7^. In Eu-activated GaN, without Mg, Eu_Ga_ association with an intrinsic defect changes the peak position of the principal ^5^D_0_ → ^7^F_2_ transition line from 621 nm (Eu2) to 622 nm (Eu1)^15^,^17^. Theoretical calculation suggests that the association of V_N_ with Eu_Ga_ (axial or basal pairs) results in a quite pronounced relaxation of the structure, with Eu displaced by ~0.14–0.23 Å from the crystal *c*-axis^22^. As Eu1(Mg) and Eu1 show rather similar peak positions^19^, we expect a similar crystal distortion. As we observed a complete conversion of Eu0 to Eu1(Mg), we need to consider that the hole is localized to the N atom linked with Eu. As discussed above, the crystal field splittings of ^7^F_1_ multiplets of the Eu0 defect suggests that the Mg atom, which associates with Eu_Ga_ (Eu2) to form the Eu0 defect, links through a non-axial nitrogen to the spectator Eu^3+^ ion. Now there are two possibilities: (1) the Mg atom sits at a Ga site along the c-axis (as in our model) and the DGS hole localises on a N atom axial w.r.t. to the Mg atom; (2) the Mg atom sits in a plane perpendicular to the c-axis and the hole localises on a N atom non-axial (basal) w.r.t. the Mg atom. In the second case, since there would be equal probability that hole localises to any of the three non-axial N atoms linked to Mg, the probability of an Eu0 to Eu1(Mg) transition would be 1/3. Since we observe a complete conversion of Eu0 to Eu1(Mg), we can easily discount this second possibility.

We conclude that in GaN(Mg):Eu at low temperatures the DGS hole is localized on a N atom that is axial to Mg. Our findings support the claim of Lany and Zunger^7^, Callsen *et al*.^9^ and Davies^10^ that there *are both* STS and DGS acceptor states in Mg-activated GaN, but it does not support their claim that in DGS the hole is localized on a basal N atom^7^,^10^. An axial hole assignment emerges in more recent theoretical studies by Lyons *et al*.^8^, Buckeridge *et al*.^12^ and Miceli and Pasquarello^13^. It is important to note further that Callsen *et al*.^9^ reported a third acceptor state ABX2 (ascribed by them to an unknown impurity) which may bear the same ‘synonymity’ to the resonance peak in our experiment as the ABX1 and ABX3 lines bear to Eu0 (STS) and Eu1(Mg) (DGS).

In summary, our results are compatible with the existence of STS and DGS acceptor states in Mg-doped GaN. The findings of this and previous work suggest that the hole in DGS is localized in an axial Mg-N bond. The activation energy for the Eu0 to Eu1(Mg) transformation is found to be ~27.7 meV, very close to the crossover energy barrier (20 meV) estimated for the STS to DGS transition in the L-Z model. Our work also provides a dynamic way of looking at the photochromic transition between configurations of the same dominant defect in doubly doped GaN(Mg):Eu. It indicates that the Eu0 to Eu1(Mg) transition rate can be effectively tuned by changing temperature and excitation density. Now we can look forward to studying the role of excitation wavelength on Eu0 to Eu1(Mg), hence STS to DGS, transformation. We will also study the reverse transformation of Eu1(Mg) to Eu0 during the warming half-cycle of the hysteresis loop, in order to seek more information regarding the hole ionisation process from the DGS and to deepen understanding of various metastable states of GaN(Mg):Eu which have great potential academic and technological significance.

## Methods

### Sample preparation

A GaN layer, 2 μm thick, grown on a 2-inch sapphire wafer by metalorganic vapour phase epitaxy, was doped *in-situ* to a concentration of 1.1–1.2 × 10^19^ Mg cm^−3^, as verified by secondary ion mass spectrometry. The wafer was sectioned into 1 cm^2^ samples and implanted with various fluences of Eu (8 × 10^12^ cm^−2^@70 keV, 1.7 × 10^13^ cm^−2^@150 keV and 6 × 10^13^ cm^−2^@380 keV) along the surface normal, in order to produce an approximately uniform Eu concentration of 1 × 10^19^at/cm^3^ from 20 to 75 nm below the sample surface. The sample was annealed at high temperature (1673 K) and high pressure (1 GPa of N_2_) to repair crystal damage created during implantation, to activate Eu-related emission and perhaps also to *generate* Eu0 centers. During annealing, the sample surface was covered with bulk GaN crystallites to prevent out-diffusion of nitrogen.

### Optical measurements

PL measurements were carried out in a closed-cycle helium cryostat (base temperature 12.5 K) with sample excitation by a 355 nm CW laser (with maximum output of 20 mW in a 1.5 mm spot), which is shorter than the absorption edge even at low temperature. The incident light intensity was controlled by inserting calibrated neutral density filters in the beam path. Sample luminescence was dispersed by a ⅔-m spectrometer and recorded using a cooled 1024 × 127 pixel CCD camera.

## Additional Information

**How to cite this article**: Singh, A. K. *et al*. Hysteretic photochromic switching of Eu-Mg defects in GaN links the shallow transient and deep ground states of the Mg acceptor. *Sci. Rep.* **7**, 41982; doi: 10.1038/srep41982 (2017).

**Publisher's note:** Springer Nature remains neutral with regard to jurisdictional claims in published maps and institutional affiliations.
